# Microfluidically supported characterization of responses of *Rhodococcus erythropolis* strains isolated from different soils on Cu-, Ni-, and Co-stress

**DOI:** 10.1007/s42770-021-00495-2

**Published:** 2021-05-06

**Authors:** Jialan Cao, Charmi Chande, Franziska Kalensee, Tim Schüler, Michael Köhler

**Affiliations:** 1 Institute for Micro- and Nanotechnologies/Institute for Chemistry and Biotechnique, Department of Physical Chemistry and Microreaction Technology, Technical University Ilmenau, PF 10 05 65, 98684 Ilmenau, Germany; 2 Department of Chemical and Materials Engineering, New Jersey Institute of Technology, Newark, NJ 07102 USA; 3 Thuringian State Office for Monument Preservation and Archeology (TLDA), Humboldtstr.11, 99423 Weimar, Germany

**Keywords:** Droplet-based microfluidics, Dose-response, *Rhodococcus*, Heavy metals, Intraspecies differences, Copper mining

## Abstract

We present a new methodological approach for the assessment of the susceptibility of *Rhodococcus erythropolis* strains from specific sampling sites in response to increasing heavy metal concentration (Cu^2+^, Ni^2+^, and Co^2+^) using the droplet-based microfluid technique. All isolates belong to the species *R. erythropolis* identified by Sanger sequencing of the 16S rRNA. The tiny step-wise variation of metal concentrations from zero to the lower mM range in 500 nL droplets not only provided accurate data for critical metal ion concentrations but also resulted in a detailed visualization of the concentration-dependent response of bacterial growth and autofluorescence activity. As a result, some of the isolates showed similar characteristics in heavy metal tolerance against Cu^2+^, Ni^2+^, and Co^2+^. However, significantly different heavy metal tolerances were found for other strains. Surprisingly, samples from the surface soil of ancient copper mining areas supplied mostly strains with a moderate sensitivity to Cu^2+^, Ni^2+^, and Co^2+^, but in contrast, a soil sample from an excavation site of a medieval city that had been covered for about eight centuries showed an extremely high tolerance against cobalt ion (up to 36 mM). The differences among the strains not only may be regarded as results of adaptation to the different environmental conditions faced by the strains in nature but also seem to be related to ancient human activities and temporal partial decoupling of soil elements from the surface. This investigation confirmed that microfluidic screening offers empirical characterization of properties from same species which has been isolated from sites known to have different human activities in the past.

**Supplementary Information:**

The online version contains supplementary material available at 10.1007/s42770-021-00495-2.

## Introduction

Closed-loop processes are one of the most important challenges for a sustainable material management. The conversion of toxic substances into environmental-compatible species is an important step in the process chain from weed out of technical products to the re-feeding into natural biological systems [1]. Microorganisms are the main players in these conversion processes [2]. It is to assume that in future, such biotechnological conversions will completely substitute high-temperature treatment and other chemical processes for material conversion.

Among the numerous of microorganisms, the genus *Rhodococcus* has attracted a lot of interest during the last decades due to the ability of several strains for bioremediation of phenol-, dye-, fuel-, or solvent-contaminated soils [3–6]. The genus is strictly aerobic and heterotroph. It belongs to the family *Nocardiaceae* in the phylum *Actinobacteria*. Bacteria as *R. erythropolis* can promote the bioremediation by direct utilization of organic contaminants as well as by supporting the bioflocculation process [7]. The *R. erythropolis* strain IBBPol, for example, is able to utilize not only aliphatic compounds as n-hexane, cyclohexane, and decane but also aromatic solvents and monomers as toluene, ethylbenzene, and styrene [8]. Thus, *Rhodococcus* is of interest for the degradation of crude oil [9], is usable for the desulfurization of fuels [10], and can also play an important role in the bio-recycling of lignocellulose, which is very crucial for the usage of renewable materials [11]. There are known *Rhodococcus* strains which are able to grow under extreme pH conditions or high salt content [12]. On the other hand, several *R. erythropolis* strains can tolerate and accumulate metal ions [13], among them chromate [14] and other toxic metals such as Zn and Cd [15] which could be involved in metalloprotein with biocatalytic properties. Furthermore, *Rhodococcus* can be applied for the production of biosurfactants [16, 17] and for the transformation of terpenes usable in the production of fragrances [18] and in the modification of steroids, which is important for drug production [19].

There is an urgent need to develop new and more efficient methods for the search of new bacterial strains with new remediation features and biosynthesis capabilities [20]. Beside the isolation of new strains, the investigation on tolerances against toxic organic substances and metals are promising directions for future screening processes. Thus, the ability of microorganisms to accumulate metals and to use the metals as co-factor of enzymes (e.g., superoxide dismutase) to degrade pollutants can be characterized. Microfluidic techniques are promising tools in order to overcome this multiparameter screening challenge. The key issue of the application of microfluidic for the cultivation of microorganisms is miniaturization, the separation of closed cultivation compartments and the realization of a high-throughput screenings. Furthermore, miniaturized microfluidic systems provide better precision in generating multiple concentrations and low sample volume for operation (sub-microliter or nanoliter range) compared to conventional macroscopic culture systems such as microtiter plates and Petri dishes. The determination of highly resolved dose-response functions is an attractive strategy compared to traditional studies in which only a few larger intervals of concentration steps can be investigated [21]. Among the microfluidic techniques, the technique of micro segmented flow [22] is well suited for an efficient characterization of microorganisms [23], heavy metal-tolerant soil bacterial [24], soil microbial communities [25, 26], as well as for eukaryotic [27] and multicellular microorganisms [28].

The co-contamination of soils by organic components and heavy metals on the one hand and essential functions of metals and metal accumulation for the synthesis and operation of special enzymes, on the other hand, makes the evaluation of metal tolerances of *Rhodococcus* strains particularly interesting [29]. In this work, micro segmented flow technique was implemented to evaluate the heavy metal tolerance of ten strains of *R. erythropolis* isolated from eight soil samples, whereby seven were taken from an ancient mining area and one probe taken from a metal-contaminated archeological site. In contrast to other microfluidic techniques, the method of micro segmented flow combines a strict separation of small cultivation volumes — typically in the nL and sub-μL range — with an accurate automatic variation of effector concentrations in small concentration steps. At the same time, a safe storing of the different cultivation volumes in the original order is provided. This allows a reliable addressing of individual effector concentrations in the single droplets and keeps the information about this concentration over the complete incubation phase. The method supplies highly resolved dose-response functions from microfluidic screenings. Here, we investigate their applicability and potential for distinguishing different strains of the same species by their sensitivity against copper, cobalt, and nickel.

## Materials and methods

Declaration: the authors declare that all procedures of these studies were performed according to the laws enforced in Germany.

### Chemicals

For the preselection cultivation, three different culture media, actinimyceten minimal medium (AM), soil extract medium (SEM), and VL55 medium, were utilized. The AM minimal medium consisted of 0.5 g/L asparagine, 0.5 g/L K_2_HPO_4_, 0.2 g/L MgSO_4_·7H_2_O, 0.01 g/L FeSO_4_·7H_2_O, and 10 g/L glucose monohydrate. The pH value of the AM media was adjusted to pH 6.8. The SEM medium was prepared as followed: 1000 ml distilled water was added with 666 g of air-dried soil and allowed to sediment for 3 h at room temperature. Thereafter, the supernatant was centrifuged at 1920×*g* for 5 min and passed through a 0.22 μm filter. Followed by 0.5 g/L asparagine, 0.5 g/L K_2_HPO_4_, 0.2 g/L MgSO_4_·7H_2_O, 0.01 g/L FeSO_4_·7H_2_O, and 10 g/L glucose were added to the soil extract. The VL55 medium consisted of 1.95 g/L 2-morpholino ethanesulfonic acid, 49.3 mg/L MgSO_4_·7H_2_O, 44.1 mg/L CaCl_2_·2H_2_O, 26.4 mg/L (NH_4_)_2_HPO_4_, 1 ml/L selenite-tungstate solution (0.5 g/L NaOH, 3 mg/L Na_2_SeO_3_·5H_2_O, 4 mg/L Na_2_WO_4_·2H_2_O), 1 ml/L trace element solution (10 ml/L HCl 25 wt%, 1.5 g/L FeCl_2_·4H_2_O, 104 mg/L CoCl_2_, 82 mg/L MnCl_2_·2H_2_O, 70 mg/L ZnCl_2_, 6 mg/L H_3_BO_3_, 36 mg/L Na_2_MoO_4_·2H_2_O, 13 mg/L NiCl_2_, 1.58 mg/L CuCl_2_), 3 ml/L vitamin solution (17 mg/L vitamin B12, 13 mg/L 4-aminibenzoate, 3 mg/L biotin, 33 mg/L nicotinic acid, 17 mg/L hemicalcium D-pantothenate, 50 mg/L pyridoxamine-HCl, 33 mg/L thiamine-HCl·2H_2_O, 10 mg/L D,L-6,8-thiotic acid, 10 mg/L riboflavin, 4 mg/L folic acid), and 0.36 g/L glucose. The pH value of the VL55 media was adjusted to pH 5.5. To obtain pure cultures of single bacterial strains, AM agar, soil extract agar (medium content and 20 g/L agar), and VL55 agar (3% washed agar) were used for the isolation. The 3% washed agar was prepared as follows: suspended 33 g agar in 2 L distilled water and stirred with a magnetic stirrer for about 5 min. Then switch off the stirrer and allowed the agar to settle for 30 min. Decanted off the supernatant and repeated this washing step about 5 times to remove sugar etc. from the agar. Finally, the desired amount of agar was resuspended in 1000 ml of water and autoclaved.

The following chemicals were utilized as effector for the preselection- and microfluid screening experiments: CuSO_4_·6H_2_O, NiSO_4_·6H_2_O, Co(NO_3_)_2_·6H_2_O, NaOH, Na_3_VO_4_, (NH_4_)_2_CrO_7_ (Merck, Darmstadt, Germany), and NaCl (VWR, Germany). Eukaryotic translation inhibitor cycloheximide was obtained from BioChemica (Düsseldorf, Germany) in order to prevent growth of soil-derived fungi in droplets. Throughout the dose-response screening experiment, the AM medium was used for the incubation of all isolates. Perfluoromethyldecalin (PP9, F2 Chemicals, Lancashire, UK) was applied as carrier medium for the separation of droplets.

### Soil sampling

Seven soil samples (E18, E27, E81, E83 E88, Q42, and B12) used in this study were collected from ancient copper mining areas and one sample (HBP4) from an archeological excavation. All the sample sites were located in the region of Thuringia and Saxony-Anhalt. The exact locations and a brief description of the soil samples are listed in Table 1.

**Table 1:** Soil sample description

Sample no.	Location	GPS coordinates (Gauss-Krueger)	Description	Collection date
E18	Hergisdorf	4463,996/5711,089	Historic mining area	24.Mar.2018
E27	Hettstedt	4466,585/5723,423	Historic copper mine	24.Mar.2014
E81	Eisleben-Oberhütte	4468,648/5712,696	Melting place	09.May.2016
E83	Eisleben-Oberhütte	4468,594/5712,73	Melting place	09.May.2016
E88	Eisleben-Oberhütte	4469,29/5711,667	Copper mine	09.May.2016
HBP4	Altenburg	4530,555/5649,797	Archeological excavation	15.Aug.2017
B12	Römhild-Steinsburg	4399,713/5587,157	Prehistoric hillfort settlement	02.May.2017
Q42	Uftrungen	4431,72/5707,443	Historic mining area	30.Dec.2015

The archeological soil sample was taken from a medieval waste pit supplying non-ferrous metal artifacts, probably related to a late medieval metal craftsman. Other soil samples (except soil sample HBP4 from the archeological excavation) were taken from the surface of the earth using a sterile falcon tube. Macro fibers and stones were removed and the probes were immediately sealed. Followed by air-drying under sterile conditions (Petri dish) at room temperature to remove excess moisture and water, 1 g soil was mixed with 15 ml distilled water and vortexed thoroughly. After centrifugation at 200×*g* for 20 min, the supernatant was passed through a filter paper to remove excess soil particles and retain bacterial spores and vegetative bacteria. Cycloheximide with a final concentration of 75 mg/L was added to prevent the growth of soil-derived fungi in droplets.

### Cultivation and identification of isolates

**Fig. 1:**
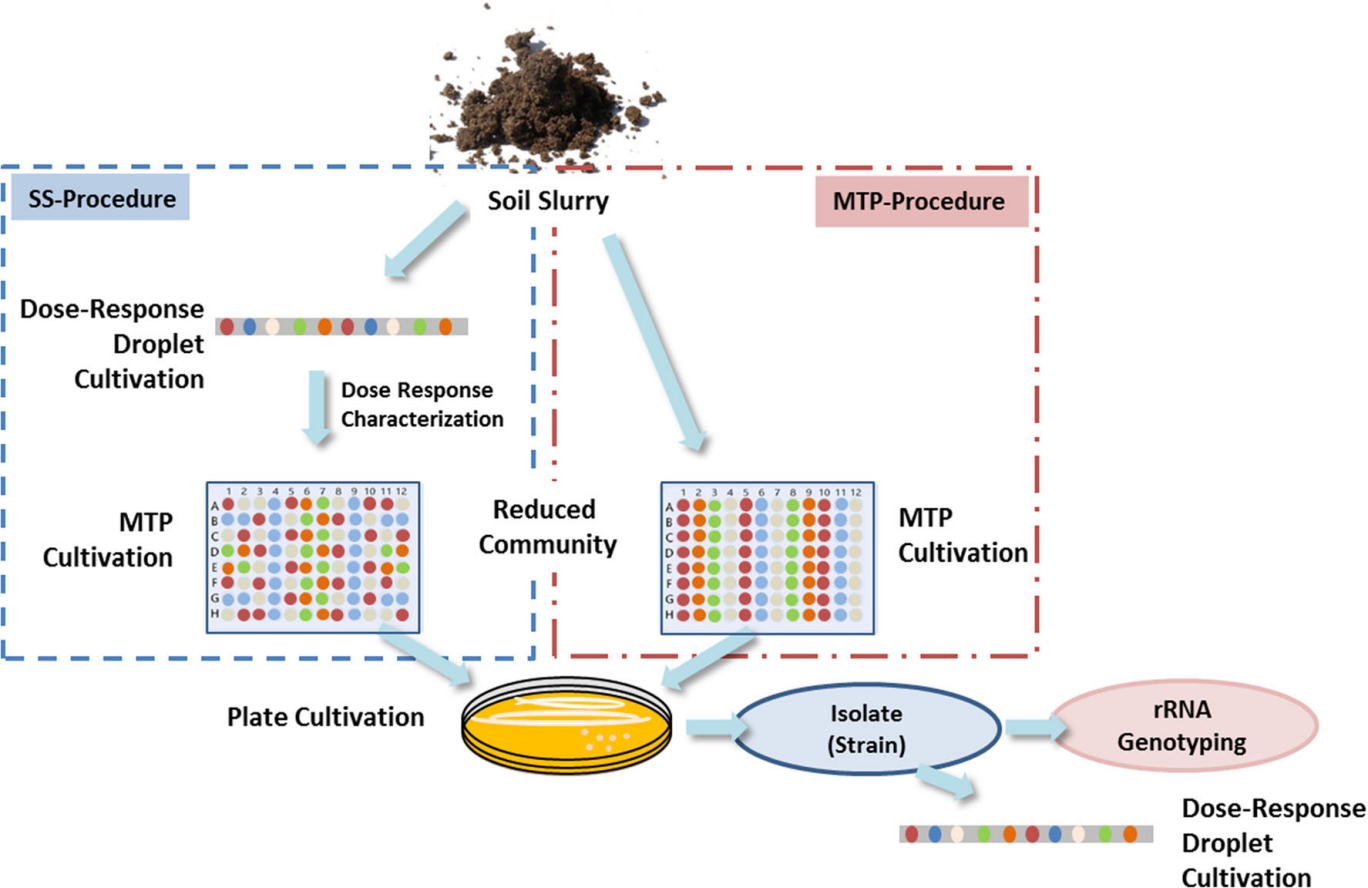
Selection strategies for identifying heavy metal-tolerant bacteria: droplet-based selection method (SS procedure) and microtiter plate-based procedure (MTP procedure)

The isolates of *R. erythropolis* from our laboratory collection were used in experiments to determine the tolerance against Cu^2+^, Ni^2+^, and Co^2+^. *R. erythropolis* were isolated from various soil samples (see Table 1) with either the droplet-based selection procedure (SS procedure) or the MTP-based diversity exploitation procedure (MTP procedure), which was already described in our previous work [30] (see Fig. 1).

For the preselection SEM, AM and VL55 media with various additions were utilized (detailed information is shown in Table 2). After a defined cultivation time, the reduced communities were applied on agar plates using the streak plate method in order to isolate single bacterial strains. The obtained cultures were characterized by Sanger sequencing after re-cultivation and successful isolation. The procedure for sample preparation for Sanger sequencing and sequence data analysis was describe in our previous work [30]. We defined that for identification on species level, a percent match of at least 99% similarity and for identification on genus level, a percent match of at least 97% are required. The isolate overview is given in Table 2. Finally, these isolates were characterized by highly resolved dose-response screenings against Cu^2+^, Ni^2+^, and Co^2+^ using microfluid technique.

**Table 2:** Isolates overview

Isolate no.	Selection procedure	Soil sample	Selection medium	Addition	Conc. of addition	% Match	Total nucleotides
F08	SS#2	E27	Soil extract	NiSO_4_	2 mM	99.93	1341
F32	SS#2	E81	AM	Co(NO_3_)_2_	0.5 mM	100	1209
F54	SS#2	E27	AM	NiSO_4_	1 mM	100	1318
F56	SS#5	hBP4	Soil extract	Co(NO_3_)_2_	1.5 mM	100	1319
F66	MTP1	Q42	AM	Na_3_VO_4_	1 mM	99.92	1332
F143	MTP3	E83	VL55	NaCl	500 mM	99.92	1219
F146	MTP3	E88	VL55	NaOH	20 mM	99.93	1356
F150	MTP3	E88	VL55	Na_3_VO_4_	1 mM	99.45	1280
F165	MTP3	E18	VL55	Na_3_VO_4_	1 mM	100	1059
F357	MTP7	B12	AM	(NH_4_)_2_CrO_7_	0.1 mM	100	1272

### Microfluidic arrangement

**Fig. 2:**
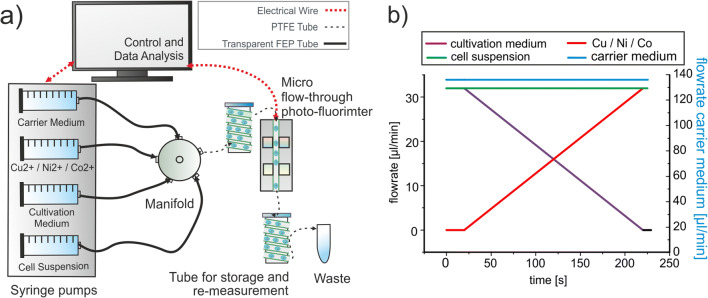
**a** Microfluidic setup for highly resolved dose-response screenings. **b** LabVIEW control program for the generation of concentration gradients

Details on the fluidic devices, the optical micro devices, and the applied methods for realizing concentration programs as well as achievable accuracy of concentration setting and calculation of the concentrations were reported earlier [31]. Here, a similar experimental setup (Fig. 2) was used for highly resolved concentration-dependent screening of effectors based on a syringe pump with four dosing units (Cetoni GmbH). The generation of the droplets was realized through a Peek™ 7-port manifold (Upchurch Scientific, USA) by controlled dosing of effectors, culture medium, and cell suspension into a flow of carrier medium (perfluoromethyldecalin PP9). The flow rate of the fluid was controlled using a LabVIEW™ program (National Instruments) for the adjustment of concentrations inside the droplets. To investigate the dose-response relationships for single substances, a LabVIEW program with continuous change of the effector concentration was applied (Fig. 2b). The first 20 s of the control program was set as positive control, followed by a 200 s concentration gradient program and ended with 5 s program with 100% of the effector concentration. The flow rates of the carrier liquid and the cell suspension were set at 136 and 32 μL/min. An increasing amount of effector (flow rate from 0 to 32 μL/min) was compensated by a decreasing amount of cultivation medium (flow rate from 32 to 0 μL/min). Therefore, the overall flow rate of the droplet generation process was kept constant at 200 μL/min. An initial cell density of 1000 cells per 500 nL droplet was applied.

The response of the different bacterial strains was characterized by their growth behavior inside the sub-microliter droplets containing the nutrition solution with step-wise varied metal ion concentrations (effectors). The cell densities bevor and after incubation period were determined by microphotometric measurements. Therefore, four microphotometers operated at four different wavelengths have been applied in order to distinguish a possible change in spectral absorption and to separate it from light scattering due to an increase of cell density by growth. In addition, a microfluorimetry was applied in order to detect changes in physiological activity by the cellular autofluorescence and to check stimulating effects of increasing metal concentration in the sub-lethal range on the production of fluorescing metabolites. The evidence of growth during incubation and its intensity was identified by comparing the photometric as well as the fluorometric signals from each individual droplet before and after incubation. Every decrease in transmitted light and every increase in fluorescence intensity indicate bacterial growth. No changes of these signals in case of high metal ion concentrations indicate the suppression of bacterial growth.

For the optical measurements of complete sequences, each droplet is transported with constant flow rate through the optical detector unit transported with constant flow rate through an optical detector unit containing flow-through photometer and fluorometer, which measures the droplet directly through the FEP tubing (Fig. 2a). Four light emitting diodes (Agilent, USA) with peak wavelengths of 470, 505, 615, and 660 nm with a spectral half-width about 30 nm were used for the scattering measurement. For monitoring bacterial autofluorescence, a laser diode with a peak wavelength of 405 nm with a bandwidth of ±5 nm (Changchun New Industries Optoelectronis, China) was used with a combination of a short-pass (455 nm) and long-pass filters (510 nm) (Laser Components, Germany). The emitted photons were counted by photomultiplier modules (Hamamatsu, Japan). The tube coils with rolled Teflon tubes (PTFE) with a length of 4 m (0.5 mm ID, 1.0 mm OD) were used to store and to incubate (at 22 °C) the generated sequences. In total, the experiments were repeated two times and for each sequence, we analyzed about 350 droplets. In addition, negative controls (droplet sequence against effector without cells) were always performed in parallel to include them for obtaining optical reference measurements for each experimental run.

## Results and discussions

The experimental concept is based on the droplet-based microfluid technology. Between several hundred and a few thousand of droplets are generated in a well-defined sequential order inside a micro PTEF or FEP tube. The input is represented by a sequence of droplets each containing the same cell density but different concentration of the effectors to be investigated. The response of cell cultures inside droplets was evaluated by two independent optical measurements, which supplies two sets of complementary information about the evolution and activity of bacteria. The total of final cell number is well reflected by the reduction of the intensity of transmitted light by use of micro flow-through photometer. This method gives information on the cumulative number of cell divisions, but it does not allow to distinguish between vital and dead cells. A certain measure for the viability is given by the endogenous cellular autofluorescence activity cause of a micro flow-through fluorimeter. In principle, different molecular species can contribute to the autofluorescence of bacteria. Different species are marked by different features of fluorescence spectra which can be used for empirically distinguishing them [32]. Currently, it is not possible to draw a closed picture of the causality of heavy metal stress on the autofluorescence response of bacterial strains. Nevertheless, two mechanisms for the influence of metals on autofluorescence are conceivable. One is the inclusion of metal ions into fluorophores — for example by complex formation — which leads to enhanced spectral absorption fluorescence quantum yields. Another mechanism is the induction of molecular stress and the increased concentration of stress-indicating molecules with fluorescence activity. In principle, a significant contributions to autofluorescence could originate from tryptophan or from NAD(P)H [33], but this fluorescence is excited in UV, because the absorption in the visible range can be neglected. Among the candidates for enhanced autofluorescence by visible light, flavin and its compounds are the most probable molecular species. These compounds play an important role in redox processes, in detoxification, as well as in the generation and reduction of oxidative stress [34]. The emission maxima around 500 nm are in good agreement with the enhanced autofluorescence intensities detected in case of sub-lethal stress in the cultivation experiments and could be interpreted as metal ion-induced increase in flavin species. Obviously, this reaction on the heavy metal stress is not only dependent on type and concentration of metal ions but also varies from strain to strain within the one investigated species.

Microorganisms that occur in biotopes with elevated heavy metal concentrations often have special resistance mechanisms to the respective metals that prevent or significantly reduce the damage caused by the toxic element. The possible mechanisms of heavy metal resistance are: (i) complexation of heavy metal ions, i.e., to bind specifically and thus prevent heavy metal ions from binding in an uncontrolled manner to nucleic acids and proteins or contributing to the generation of free radicals [35], (ii) removal of heavy metal ions by efflux transporters [36], (iii) enzymes for detoxification of highly reactive oxygen species [37], and (iv) reduction of the toxicity of a heavy metal by changing its valence [38].

### Dose-response screening against CuSO_4_, NiSO_4_, and Co(NO_3_)_2_ of two *R. erythropolis* strains isolated from one soil sample

**Fig. 3:**
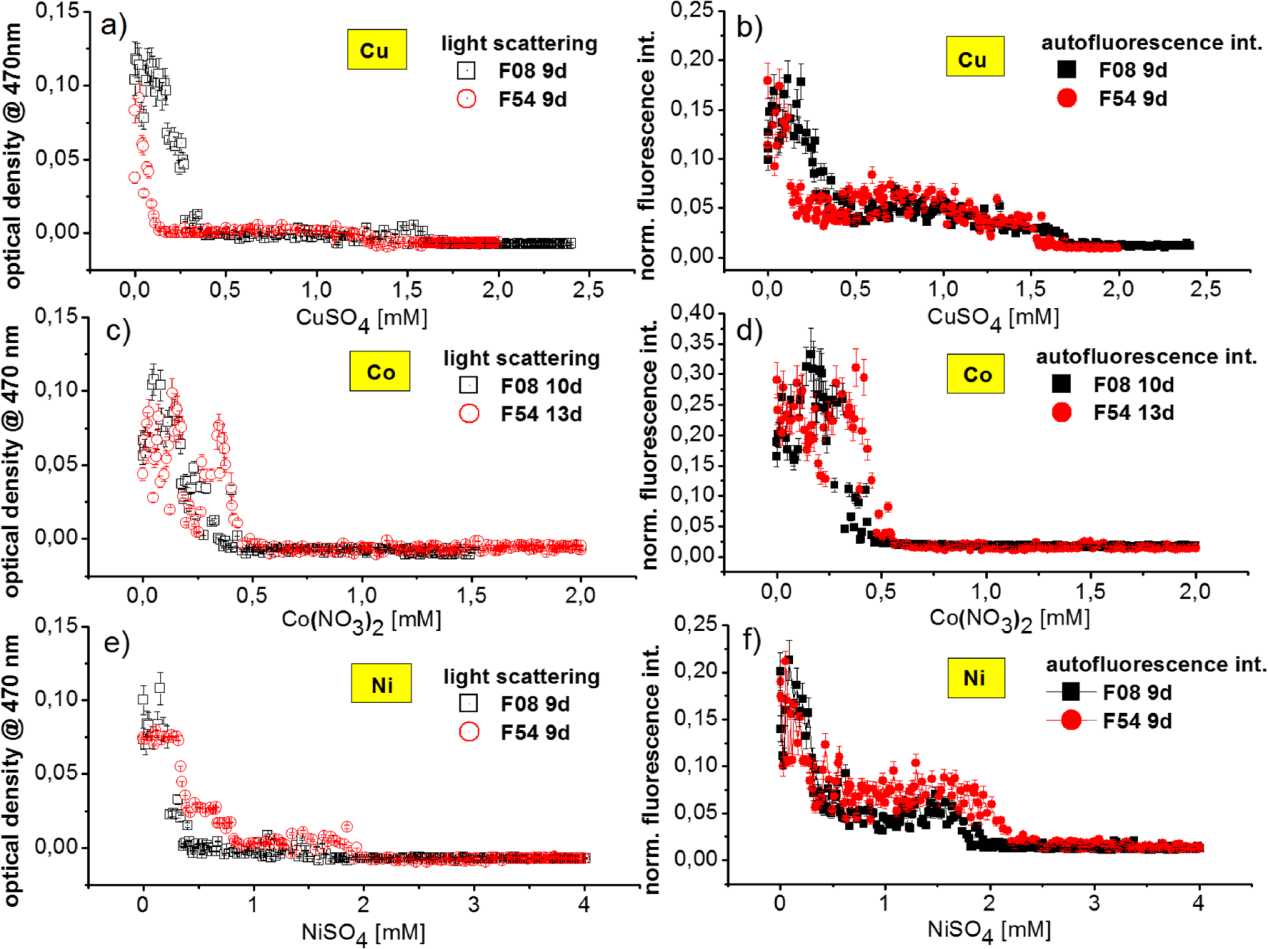
Comparison of highly concentration-resolved dose-response functions of two *R. erythropolis* isolates F08 (square), F54 (circle). Both strains were inoculated together with increasing concentrations of copper (**a**, **b**), cobalt (**c**, **d**), and nickel (**e**, **f**) into 500 nL droplets. Extinction at 470 nm excitation (**a**, **c**, **e**) and fluorescence at 405/425 nm excitation/emission (**b**, **d**, **f**) from the *R. erythropolis* in the droplets were measured by micro flow-through sensing after 9 to 13 days incubation. Every dot represents one droplet

Soil slurry from soil E27 was inoculated together with increasing concentrations of nickel and copper into droplets by using AM and soil extract medium. After 35 days incubation inside droplets, F08 was isolated from the well with soil extract medium in combination with 2 mM Ni^2+^. F54 was isolated from AM medium in combination with 1 mM nickel stress (see Table 2). Both *R. erythropolis* strains isolated from soil samples E27 were compared for their behavior with respect to addition of copper, nickel, and cobalt to the nutrition medium. The results are reflected by microphotometric (Fig. 3a, c, e) and microfluorometric measurements (Fig. 3b, d, f). Each dot in the graphs represents the optical signal of one droplet of a volume of about 0.5 μL. The measurement beam has a diameter of about 0.5 mm, which corresponds to the complete internal diameter of the micro tube. The integration of optical signal over the complete residence time of the droplet ensures the registration of a representative optical signal for each individual cultivation volume. The averaging over the entire droplet is additionally supported by the transport-induced circular droplet–internal convection of the liquid inside the droplets. All measurement values of one graph were obtained in one single experimental run, so that all droplets experienced exactly the same conditions and procedures.

As results, the optical density and the fluorescence of droplets change similarly with the increase of metal ion concentration and all isolates show a similar response for all three metal ions (Fig. 3). This speaks for the fact that both signals reflect mainly the density of cells inside single droplets and the dependency of bacterial growth on metal ion concentration. In the case of Ni^2+^, an unaffected growth at low concentrations of up to 0.3 mM and a moderate growth between 0.3 and 2.1 mM Ni^2+^ (Fig. 3e, f) was observed. This intermediate response is clearly separated from the unaffected growth at low concentrations and total inhibition at higher metal concentrations and was observed for both strains. A similar behavior to nickel was found in the presence of copper; three different types (intensive growth, limited growth, and growth inhibition) of growth responses with respect to different concentration ranges were observed for all strains. The unaffected growth of F08 was located at 0.25 mM, while F54 was found at 0.12 mM Cu^2+^. Above 0.25 mM Cu^2+^ of F08, the autofluorescence intensity was significantly reduced with only 5% intensity compared to the droplets with less than 0.25 mM Cu^2+^. The same step-wise response was also observed by F54. It was found that the critical value for Cu^2+^ of F54 (1.5 mM) was about 10% lower than F08 (1.7 mM Cu^2+^).

Much lower metal tolerance of all strains was evidenced when cobalt ions were applied. Compared to nickel and copper, with increasing cobalt concentration, a significant and sharp transition between growth and growth inhibition was found. A total inhibition at 0.5 mM Co^2+^ was determined for both isolates. While copper and nickel are essential for many different enzymes, cobalt is necessary for the function of only a limited number of enzymes and results for a generally higher sensitivity and a less developed homeostasis and storage characteristics for this metal.

The comparison of the responses of the isolates F08 and F54 on three metals indicated that both *R. erythropolis* could be originating from the same strain in the soil sample. This interpretation was in coordination with 16S rRNA results determining 99.93 and 100% similarity between the strains.

### Response during cobalt stress of *R. erythropolis* strains isolated from different soil samples

**Fig. 4:**
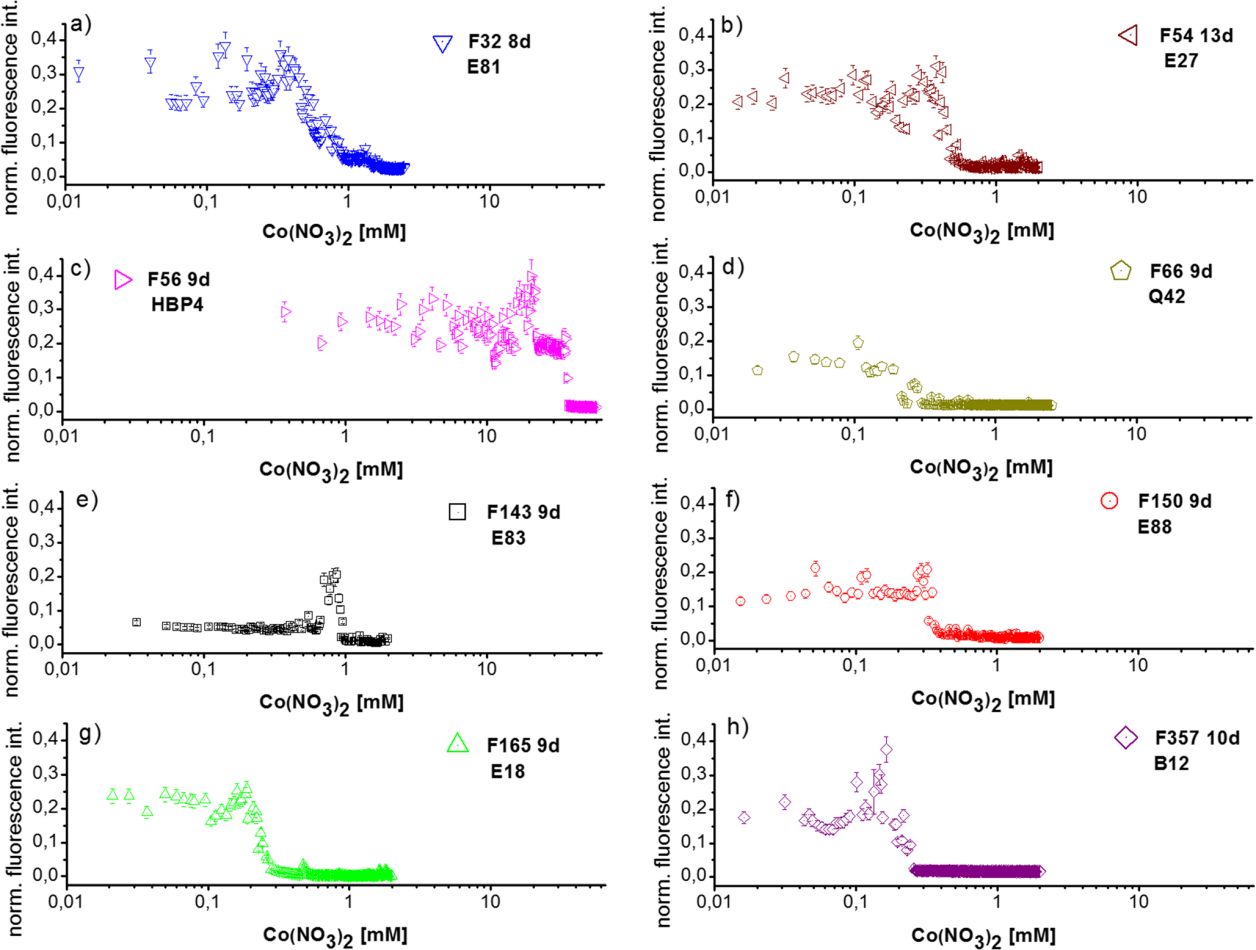
Fluorometric data from concentration-dependent microfluidic screening for the response of *R. erythropolis* isolates obtained from eight soil samples on Co^**2**+^ stress (see Tables 1 and 2 for origin of samples)

Different *R. erythropolis* isolates could be compared by their highly resolved dose-response patterns obtainable through concentration-dependent microsegmented flow cultivation. These patterns were shown for eight isolates. The growth was evaluated after 8–13 days of cultivation by measuring the autofluorescence intensity at 405 nm utilizing the microfluorometry. As a result, it was observed that all strains differed considerably in the critical cobalt concentration as well as in the average autofluorescence activity below the critical concentration (Fig. 4). Whereas F32, F54, F66, F143, F150, F165, and F357 appeared to have a comparatively low Co^2+^ tolerance (0.25-0.9 mM), the strain F56 showed a significant reduction of fluorescence only above a concentration of 36 mM Co^2+^ (Fig. 4).

The highly resolved dose-response functions of Fig. 4a show a normal growth occurred at up to 0.45 mM Co^2+^ and a total inhibition of growth was observed above 0.9 mM for the strain F32. With an increasing concentration of Co^2+^ between 0.45 and 0.9 mM, the autofluorescence intensity gradually decreased. A comparatively steep transition between full growth and suppression was observed in case of F54, F56, F150, and F165 against Co^2+^. The following strains evidenced a lower cobalt tolerance: F66 and F165 were totally inhibited at 0.3 mM, F150 at 0.35 mM, and F357 at 0.25 mM (Fig. 4d, f, g, h).

The observed low autofluorescence after the incubation indicates a low concentration of cells in the droplets in case of the isolate F143 from soil sample E83 at low cobalt concentrations. It is probably caused by a slow growth rate of the bacteria colonies inside the droplets during the incubation period. F143 was significantly stimulated at sub-lethal doses of Co^2+^ (above 0.85 mM). The autofluorescence rose up to considerably higher values (about 300%). The high resolution revealed a strict transition between this highly stimulated growth and complete suppression at a Co^2+^ concentration of 1 mM (Fig. 4e). F357 showed similar, but not identical, growth behavior. A pronounced stimulatory effect at sub-lethal doses of Co^2+^ at about 0.15 mM was observed for strain F357 (Fig. 4h).

Certain specificity was found in the sub-lethal range of strain F56. Between 0 and 20 mM Co^2+^, the fluorescence of single droplets turned out to be more scattering when the cobalt concentration increases. This behavior can be interpreted as a consequence of the stochastic confinement effect due to the small cultivation volumes. The developing bacterial populations in single droplets responded individually to the increase of cobalt concentration. Some of them show a significantly reduced autofluorescence, other responds with a considerable enhancement of fluorescence activity. The range between 20 and 35 mM is marked by a lowering in autofluorescence and a lower scattering rate on individual responses. It seems that the moderate cobalt stress leads to the differentiation of individual responses of the small start populations inside droplets. It can be summarized that F56 is a highly cobalt-tolerant strain. Bacterial cells and small populations of this strain react with an increasing spectral width of physiological activity on the increasing cobalt stress over a large concentration range.

### Correlation of Cu^2+^/Ni^2+^ and Co^2+^/Ni^2+^ tolerance as a function of the origin of the soil samples

**Fig. 5:**
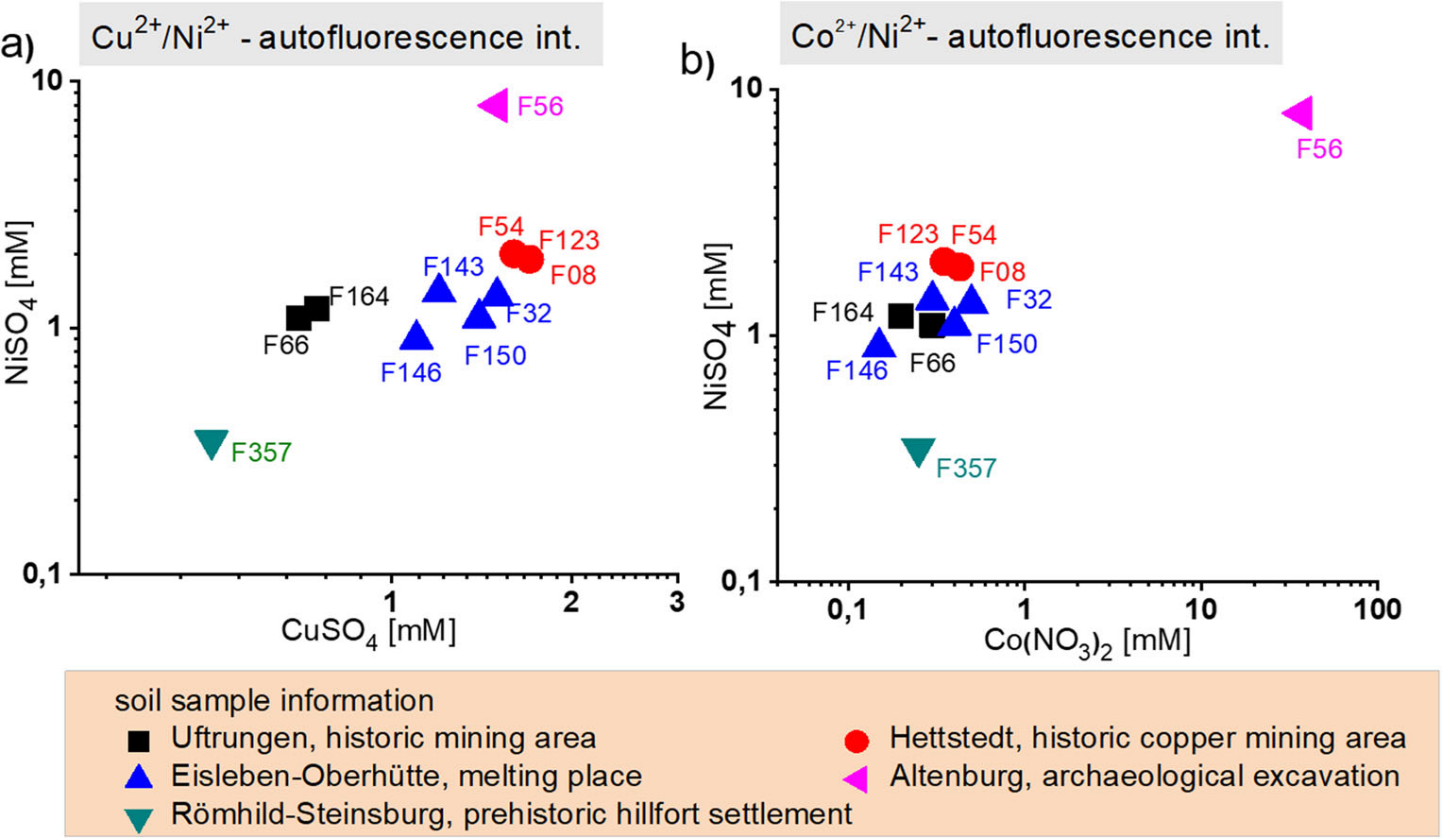
Correlation diagram of **a** Ni^**2**+^ and Cu^**2**+^ and **b** Co^**2**+^ and Ni^**2**+^ tolerance of ten *R. erythropolis* strains isolated from different origin of the soil samples. Data based on the autofluorescence intensity measured by micro flow fluorometer after 9–11 days incubation inside droplet

The correlation of the total inhibition value of all ten separately isolated *R. erythropolis* strains against CuSO_4_, NiSO_4_, and Co(NO_3_)_2_ shows a strong relation to the origin of the isolates. Despite the fact that the number of investigated samples and isolated strains are limited, the distribution of critical concentrations of the both displayed metal ions lead to remarkable differences. The narrow values for F08, F54, and F123 (red circle) confirm that the isolates are representing the same strain from soil sample E27 (see Table 2). Further, it is remarkable that the isolates F32, F143, F146, and F150 (blue triangle) are relatively close together, but less strongly connected to each other as the isolates F08, F54, and F123 from soil sample E27. These cultures have been isolated from three different soil samples, which have been taken in one area near Eisleben-Oberhütte, a former copper smelting area. F66 and F164 (black square), which came from more distant sampling sites (ancient mining places near Hergisdorf and near Uftrungen), show lower copper tolerances. The most important difference was observed between the sample from the archeological excavation (hBP4, medieval city of Altenburg, East Thuringia) and all other samples concerning the tolerance against cobalt ions (purple triangle). Whereas for all other samples critical thresholds between about 0.1 and 1 mM against Co^2+^ have been found, the isolate F56 from sample hBP4 shows a very high tolerance threshold of about 40 mM Co^2+^. At the same time, this strain is also marked by significant higher tolerance against Ni^2+^ (Fig. 5). In contrast, the isolate F357 (green triangle) is marked by a significant lower nickel tolerance in comparison with the other strains originating from the copper mining region.

The importance of the sample origins became much clear after including critical values of copper ion concentration. The comparison of copper tolerances confirms the special characteristics of the strain isolated from the Römhild-Steinsburg sample F357 (green triangle) with low Cu^2+^ and low Ni^2+^ tolerance (Fig. 5a). This specific character can be understood by the character of the place, what means from the surface of an area which is given-off as a settlement place more than 2000 years ago and now in a forest-covered protected natural area. In contrast to that, F56 is not originating from a surface sample, but from a buried cultural soil from an intensively used urban settlement area, and all other samples are originating from earlier copper mining and smelting places. These circumstances make plausible that all these isolated strains are marked by a significant higher Cu^2+^ tolerance than the strain F357. Thus, the correlation plots indicate *R. erythropolis* strains with different similarities from a phenotype point of view (Fig. 5). Obviously, the tolerance values for the different strains reflect the phenotypic divergence of *R. erythropolis* from the different places and regions.

The results are encouraging for future investigations of larger quantities of isolates from different places, on the one hand, to improve the statistical relevance of the site-dependent response of soil bacterial strains of the same species and also to pronounce the requirement of a broader screening of soils for assessing diversity in the response of soil bacterial communities with different place histories.

## Conclusions

The investigation shows that the highly resolved dose-response data obtained by microfluidic cultivation studies are well suited for characterizing the response of different strains of the soil bacterium *R. erythropolis* and for distinguishing different susceptibilities of strains of this species from different sample sites. Characteristic highly resolved dose-response functions have been obtained for all ten isolates R. *erythropolis* strains and for the three investigated metals copper, nickel, and cobalt by the microfluidic screenings. These response functions supply the metal ion concentrations for total inhibition. In addition, they reflect transitions between fast growth and reduced growth of bacteria in dependence of metal ion concentrations, in some cases. The high number of individual small test volumes in each screening run allows further to identify non-monotonous changes in the concentration-dependent growth and activity behavior as the formation of sub-lethal peaks in the dose-response functions. They are found, in particular, in the autofluorescence signals from droplets with metal concentrations in the sub-lethal range. Their appearance differs strongly for the different strains and metals.

An essential result of the investigation is the finding that differences in the heavy metal susceptibility of the various strains are related to the special local situation and differences of the character of sampling sites, although both investigated strains have nearly identical 16S rRNA sequences, which are 99.93 and 100% matching the known sequence of the species *R. erythropolis*. It can be concluded that two isolates, which had been obtained by different cultivation procedures from one soil sample (F08, F54), represent one strain, which could be concluded from the very similar observed critical metal ion concentrations. In contrast, these strains are significantly distinguishable from isolates of soil samples from other places. Particularly large differences exist between the critical concentrations of the soil samples from the ancient East Harz copper mining region and a sample from an archeological excavation of Altenburg, which was indicated from a soil which had been covered by soil and undisturbed stored since the Middle Ages. Our study showed that microfluidic screenings are useful for the empirical characterization based on the susceptibility of different strains on three selected heavy metals. Further confirms that the response of strains isolated at various sites can be distinguished from each other. However, a complete characterization of genetic and physiologic differences of isolated strains cannot solely be based on the 16S rRNA sequence similarity [39]. Thus, further phenotypic characterization and whole genome sequencing should be considered in the future.

In summary, it can be concluded that the highly resolved dose-response functions from microsegmented flow cultivation are very powerful for evaluating the responsivity of local bacteria strains of the same species against components of environmental pollution. The microfluidic strategy is very promising for empirical characterization of soil bacterial strains from special places with different human activities in the past. Furthermore, this technical approach could be utilized in the future to isolate new strains with enhanced potential for growing in contaminated areas, for bioremediation, and for characterizing the ecological value of soil organisms, in general.

## Supplementary Information

ESM 1 (DOCX 4704 kb).

## Data Availability

The data used to support the findings of this study are available from the corresponding author upon request.
