# Supplementary material for: Microfluidically supported characterization of responses of Rhodococcus erythropolis strains isolated from different soils on Cu-, Ni-, and Co-stress
Source: Braz J Microbiol. 2021 May 6;52(3):1405–15. doi: 10.1007/s42770-021-00495-2 (PMC8324611; doi:10.1007/s42770-021-00495-2)
Supplement: Supplementary file 1 — (DOCX 4704 kb). [file 42770_2021_495_MOESM1_ESM.docx]

Supplementary Information


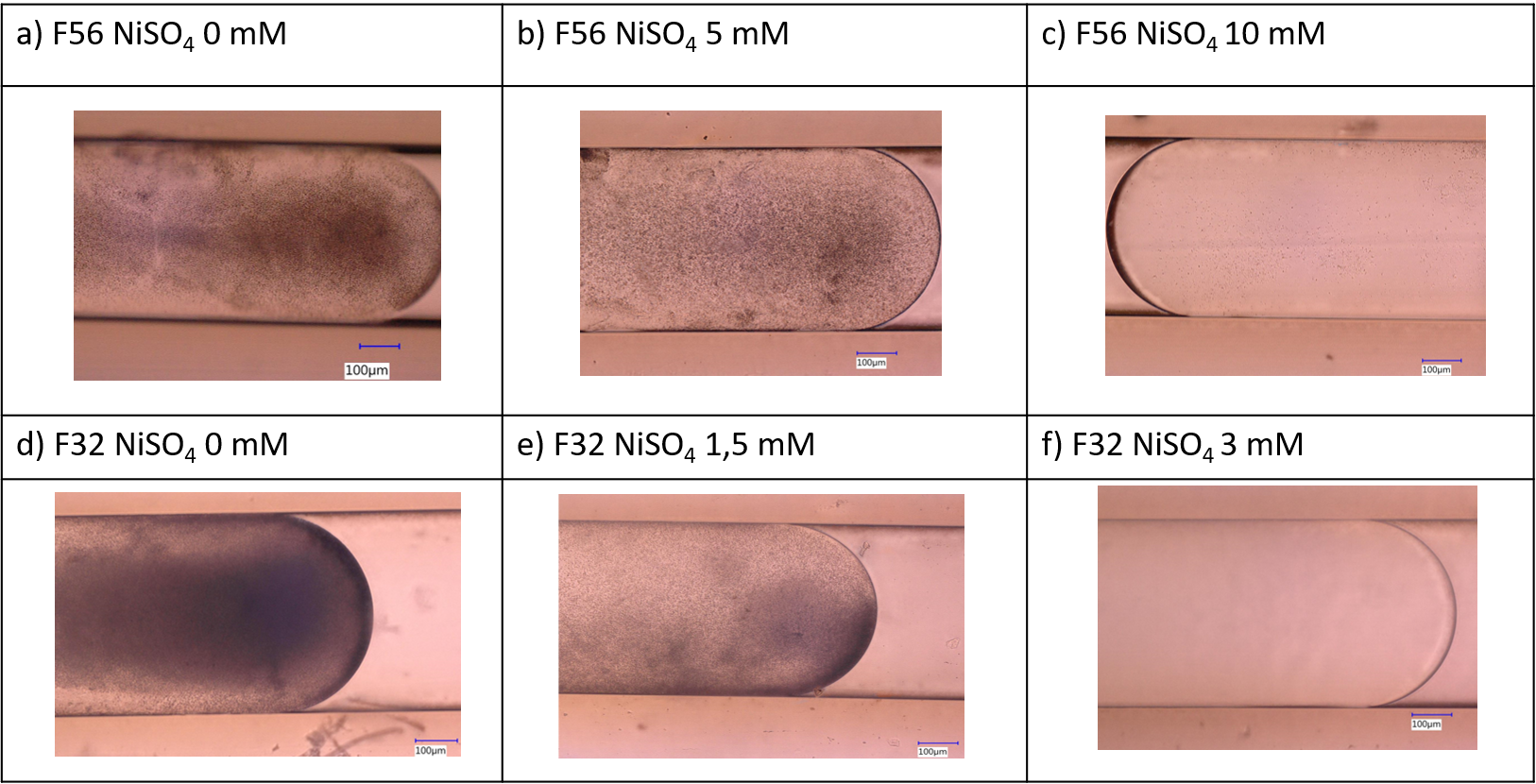


**Fig. S1: Microscopic image after 10 days incubation of *R. erythropolis* inside 500 nl droplets. of the droplets with *R. erythropolis* strain F56 (a -c) and F32 (d-f) cultivated with different concentrations of NiSO_4_**


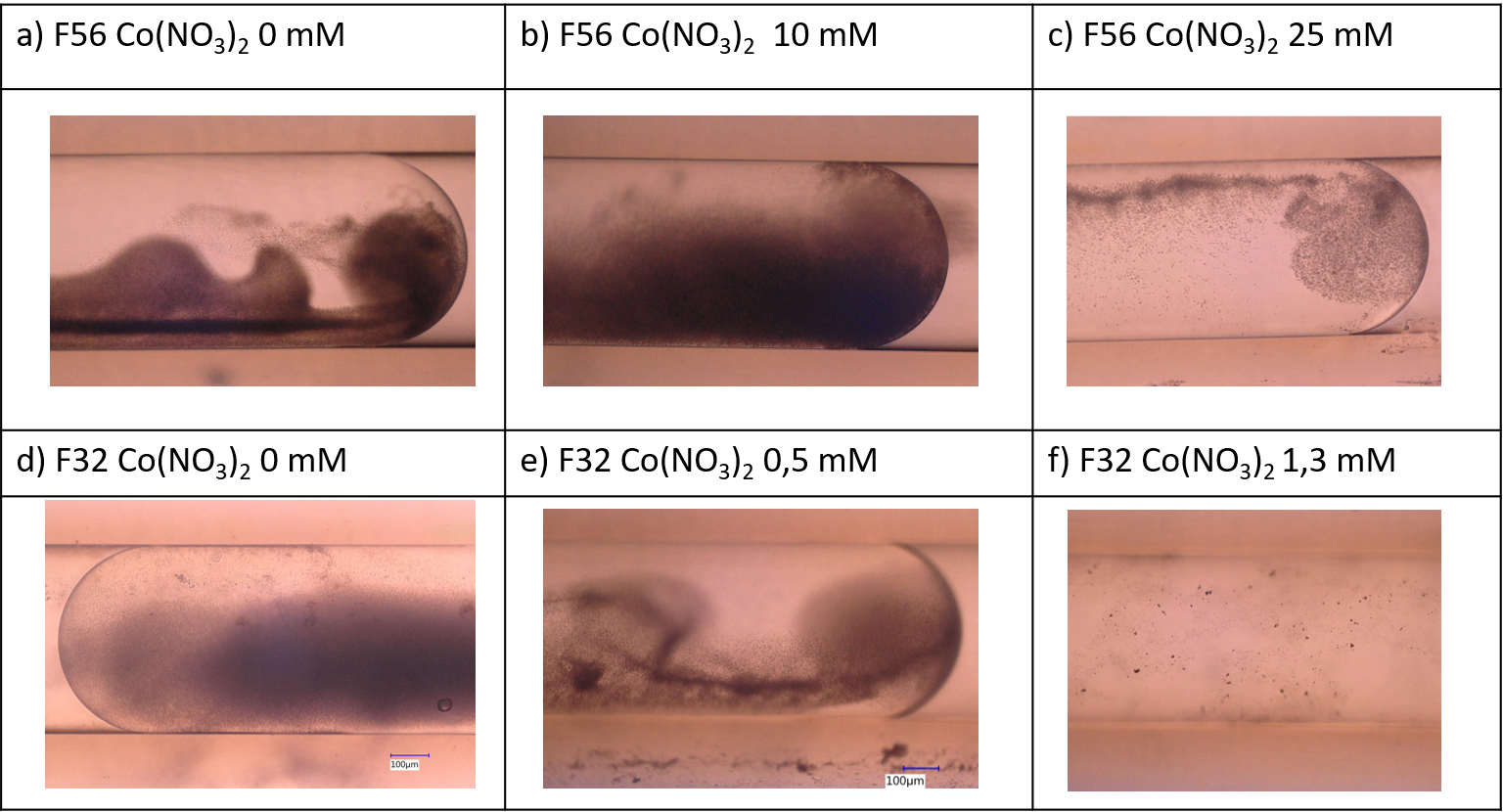


**Fig. S2: Microscopic image after 10 days incubation of *R. erythropolis* inside 500 nl droplets. of the droplets with *R. erythropolis* strain F56 (a -c) and F32 (d-f) cultivated with different concentrations of Co(NO_3_)_2_.**


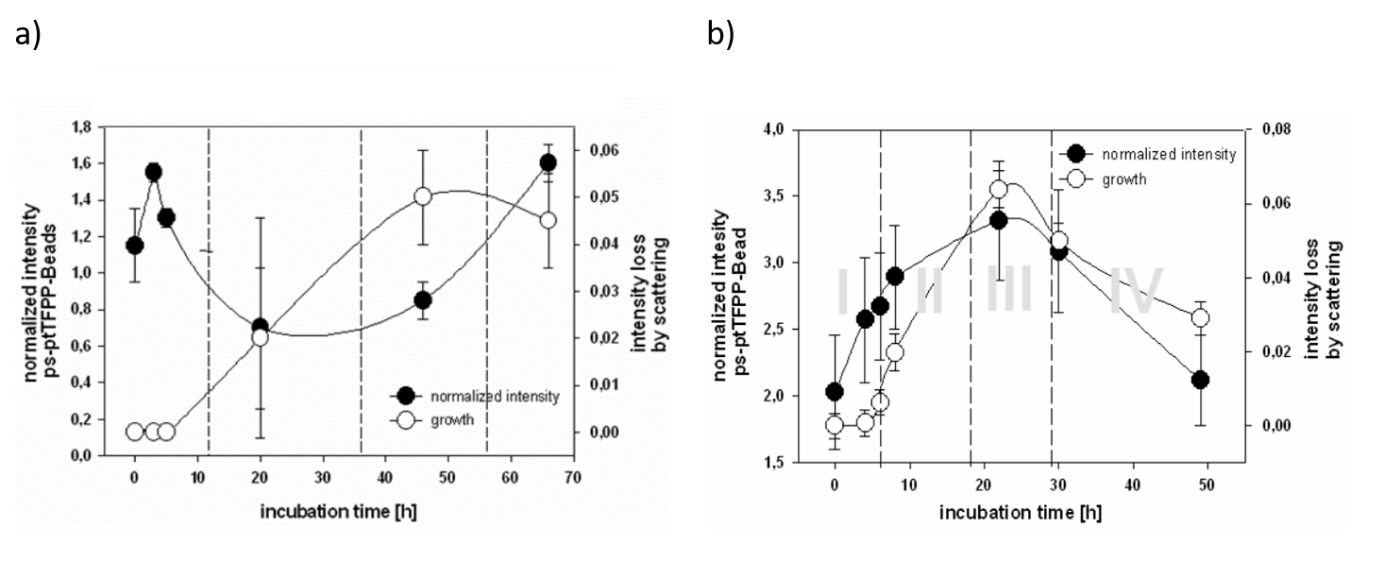


**Fig. S3: Oxygen consumption and growth curves for the *E. coli* cultivation in droplets. Inoculum cell density: 10E5 cells/mL, droplet volume: 550 nL. increased normalized intensity 🡪 oxygen consumption, intensity decrease 🡪 increase oxygen partial pressure. a) PTFE-Tubing with dw= 0.55 mm, b) PTFE-Tubing with dw= 0.25 mm.**


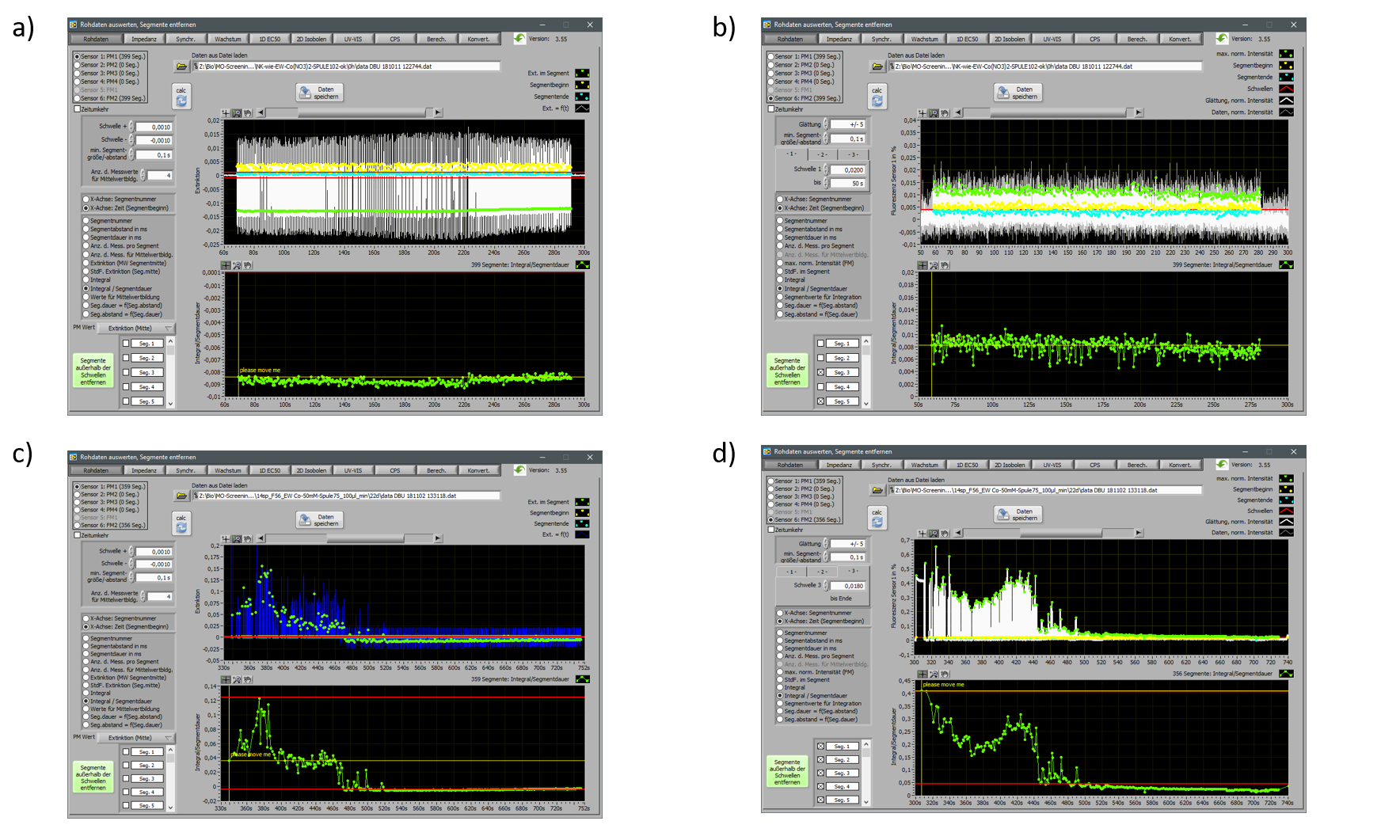


**Fig. S4: Data evaluation of the droplet sequences. Dose-Response Screening against Co(NO3)2 after 11d incubation inside droplets. A) photometric signal without cells. b) fluorometric signal without cells. c) photometric signal with cells and d) fluorometric signal with cells.**
